# Power-law scaling of calling dynamics in zebra finches

**DOI:** 10.1038/s41598-017-08389-w

**Published:** 2017-08-21

**Authors:** Shouwen Ma, Andries Ter Maat, Manfred Gahr

**Affiliations:** 1 Max Planck Institute for Ornithology, Eberhard-Gwinner-Straße, 82319 Seewiesen, Germany; 2 Graduate School of Systemic Neurosciences (GSN), Ludwig-Maximilians-Universität München, Großhaderner Str. 2, 82182 Planegg-Martinsried, Germany

## Abstract

Social mammals and birds have a rich repertoire of communication calls. Some call types are uttered rarely but in specific contexts while others are produced in large numbers but are not linked to a specific context. An example for the latter is the “stack” call that zebra finches (*Taeniopygia guttata*) utter thousands of times per day in a seemingly erratic manner. We quantified this calling activity of captive zebra finches by using on-bird telemetric microphones that permitted a precise temporal resolution. We separated the calling interactions into the reactive and the self-contained calls. Despite a large dynamic range in the succession of calling events, the temporal distribution of the reactive and the self-contained callings was characterized by a power-law with exponents ranging between 2 and 3, which implies that all calls in that scale have similar dynamic patterns. As birds underwent physiological (water availability) and social (separation from the reproductive partner) changes, their calling dynamics changed. Power-law scaling provided an accurate description of these changes, such that the calling dynamics may inform about an individual’s physiological and/or social situations state, even though a single “stack” call has no predetermined meaning.

## Introduction

Many animals, in particular group-living birds and mammals communicate with repertoires of species-specific but individualized sounds such as calls that might be uttered in very large numbers during the course of a day^1–3^. Individual calls can be uttered either with a specific context^4–10^ or without a discernable context^11, 12^. The latter have no obvious predetermined signaling value and often occur in diverse circumstances such as group progression and social interaction^11, 13, 14^. Previous works on vocal communication have focused mainly on context-specific calls. Those studies examined either the relationship between form and function of such calls^1, 10^ or the linguistic-like characteristics^15, 16^. Context-specific calls account, however, only for a small part of the emitted sounds. Some group-living birds produce thousands of calls per day that are not answered by conspecifics and that are not answers to calls from other birds^17, 18^. “Stack” calls of captive zebra finches (*Taeniopygia guttata*) for example appear to be self-contained without specific context, in particular those that are not contingent upon mates or other group-members. Both self-contained callings (own call is uttered after an own call) and reactive callings (own call is uttered after the call of others) of mammals and birds occur with a large dynamic range^19, 20^. The problems of characterizing these events in previous studies are due to the fact that the production of these calls appears not to follow any discernible pattern and has no predetermined meaning.

Insights into behavioral patterns are often gained by using mathematical models that describe complex behaviors. In a stream of vocalizations for example, some callings might be self-contained action patterns and others are reactions during vocal interactions, while both actions are performed identically in terms of muscle activity and observed movements. One method to discriminate between self-contained and reactive callings is to use a mixture model that divides the behavior into self-dynamics and pairwise reactions^21^. Moreover, traditional time series analysis mainly employs auto- and cross-correlations to analyze self-contained and reactive behaviors on the short-range of temporal correlations in bird and human dyads^17–19, 22–24^. However, similar to human activities such as email communication^25^ and mobility^26^, the “stack” calling of zebra finches has a much larger dynamic range^19, 20^. Time series analytical tools that cover large dynamic ranges (hours to several days) such as power-law distributions showed indeed that human activities follow a power-law distribution^25^. That is, the frequency of interaction between two individuals is approximated by an inverse square power exponent (*α*) of inter-event intervals of interactions^25^. The power-law description confers strong predictive power and has been used to reveal the orderly scale-free pattern in many complex behaviors^27–29^. For example, healthy heart activity in humans displays a scale-free pattern and changes of scaling exponents reflect a pathologic state^28^. We speculated that the dynamics of the self-contained and of the reactive callings might be described by power-law distributions and vary with the physiological states of the zebra finches.

Debates about the dynamics of natural behaviors concern the bursts of activities^19^ as compared to the inter-event intervals of single activities^30^. In this study, we quantified the calling activity of zebra finch pairs by using on-bird telemetric microphones that permit a high temporal precision of the measurements while the animals interact freely with their reproductive partners. We examined the statistical support^31–33^ for various mathematical models for the self-contained and reactive callings, which represent self-dynamics and reactions, respectively. Subsequently, we probed the models of self-contained and reactive callings by perturbation experiments to directly access the plasticity and homeostasis of the behaviors^21^. We applied biologically meaningful perturbations by either limiting social-sensory cues or the access to water in order to explore the dynamic changes of the reactive and self-contained calling activity^23, 34^. In many species including the zebra finch, audio-visual integration is a common feature^23, 35–37^. Hence, separating auditory from other sensory cues emitted by the mate may change the reactive calling. Furthermore, the reproductive activity of zebra finches depends on the availability of water. Previous studies showed that water-restricted zebra finches reduced their singing activity^38^ but otherwise adapted rapidly to that condition, reflecting their evolutionary origin as birds of arid zones. Hence in this study we removed water for a short period from one individual (male or female) of a mated pair at a time but allowed social (audio and visual) contacts between them. We examined dynamic changes of self-contained and reactive callings in response to the above treatments.

## Results

### Quantification of calling activity

**Figure 1:**
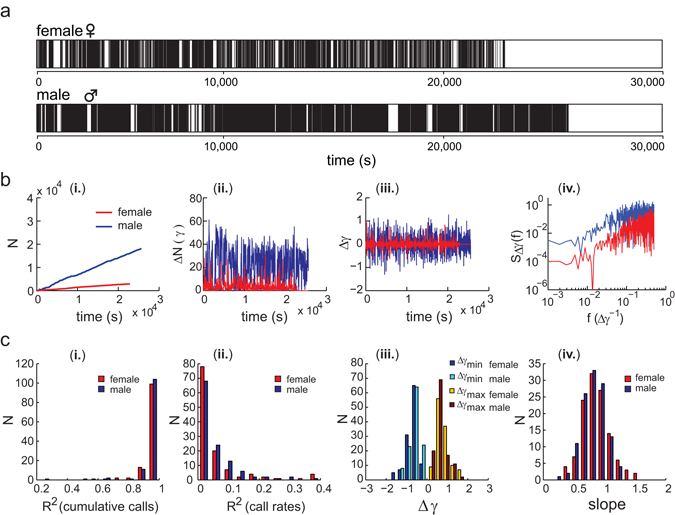
Calling activity of zebra finch pairs. (**a**) Example of a succession of calling interactions between a female (upper panel) and a male (lower panel) zebra finch. Vertical black lines corresponding to calling events are plotted as a function of time (in seconds). (**b**) Quantitative measurements of calling activity of one zebra finch pair (male in blue, female in red). (**b** [i.]) Calling events were accumulated in a linear relationship over time; (**b** [ii.]) the call rates (γ) corresponds to the number of calls (ΔN) in a unit of time (Δt = 30 s); (**b** [iii.]) The changes of call rates (Δγ) were plotted as the function of time; (**b** [iv.]) The power spectra (S) and the frequency (**f**) of Δγ were positively correlated. (**c**) Statistical summary of all 22 tested zebra finch pairs including 119 days of calling activity. (**c** [i.]) The density counts of the coefficient of determination (R^2^) of cumulative calls (bin: 0.1). The values of R^2^ are in range from 0 to 1. With increasing linearity of the data, R^2^ approaches 1. (**c** [ii.]) The density counts of the R^2^ of call rates (bin: 0.04). With decreasing linearity of the data, R^2^ approaches 0. (**c** [iii.]) The density counts of the changes of call rates (Δγ) (bin: 0.35). (**c** [iv.]) The density counts of the slopes for the power spectrum of Δγ (bin: 0.14).

To quantify the calling activity of the zebra finches, we used a telemetric audio recording system to identify the vocal events generated by 22 male-female pairs of zebra finches with high temporal precision. The vocalizations of birds were recorded continuously within the bound of one light/dark cycle. Thus, the successive calling events and the silent pauses correspond to the calling activity and the periods of inactivity, respectively, while we excluded the night period between lights off and light on (Fig. 1a & Supplementary Fig. S1a–o). For both male and female zebra finches, the numbers of calls accumulated in a nearly linear relationship with time (Fig. 1b[i.]). About 90% of measurements (108 and 106 of 119 days of measurement for males and females, respectively. 22 pairs) showed high coefficients of linear relationships (R^2^ > 0.9) between the numbers of accumulated calls and time (Fig. 1c[i.]). If we consider that the change of calling activity (ΔN) with respect to time (Δt) corresponds to the call rate (γ), we obtain:

1 $$\frac{{\rm{\Delta }}{N}_{i}}{{\rm{\Delta }}{t}_{i}}={\gamma }_{i}$$

where *i* indicates the individual male or female. The call rates (γ) of males and females were not constant over time but appeared to vary appreciably with 86% of the regression coefficients being <0.1 (Fig. 1b[ii.] and c[ii.]). Furthermore, changes in call rate (Δγ) varied in a range between −2 and 2 calls per second squared (Fig. 1b[iii.] and c[iii.]). The average Δγ_min_ are −0.66 and −0.55 and the average Δγ_max_ are 0.74 and 0.60, for females and males, respectively. The periodograms show a positive correlation between the power spectra (S) and the frequency (f) of Δγ (see example in Fig. 1b[iv.]). This indicates that positive and negative values of call rate changes are close to each other and alternate in time. The average slopes of the periodograms are 0.86 and 0.82 for female and male birds, respectively (Fig. 1c[iv.]). This analysis showed a short-range fluctuation in the changes of call rates. Subsequently, we explored the potential power-law behavior of the inter-event intervals.

### Power-law scaling of calling interactions

**Figure 2:**
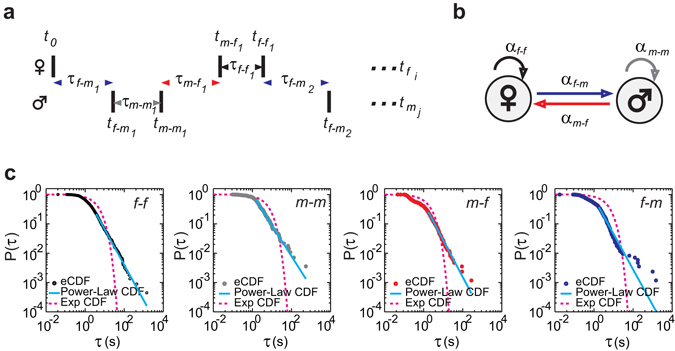
The self-contained (female: “*f-f*”; male:“*m-m*”) and the reactive (female: “*m-f*”; male: “*f-m*”) callings exhibit power-law dynamics. (**a**) Schematic of calling events (*t*) and inter-event intervals (*τ*). (**b**) Calling interactions are described as a mixture of self-contained (“*f-f*”; “*m-m*”) and reactive (“*m-f*”; “*f-m*”) callings of male and female zebra finches. (**c**) The empirical cumulative distribution functions (eCDF, dotted line, dark blue) of the inter-event intervals (*τ*) were plotted in a log-log scale. Estimated power-law cumulative distribution function (Power-Law CDF, solid lines, light blue) were modeled by using the maximum likelihood method^32^. Estimated exponential cumulative distribution functions (Exp CDF, dashed lines, magenta) were modeled with corresponding average inter-event intervals (*μ*_*τ*_). Data are shown from the female-male pair “a”. *α*: the exponent of a power-law distribution; P(*τ*): the cumulative distribution of the inter-event intervals; *τ* (s): the inter-event intervals in seconds. (for estimated parameters, see Supplementary Table S1).

It is uncertain whether the burst-like vocal activity and the inter-event intervals between single calls are distributed exponentially, log-normally or according to power-law^31, 32^. To study the dynamics of calling interactions, we considered a model consisting of self-contained and reactive callings to describe calling interactions. For a fixed recording time window [0, T], let the initial event be *t*_0_ at 0, the times of calling events for female and male be *t*_*f* i_ and *t*_*m* j_, respectively. Self-contained events follow vocal events of the same bird (*t*_*f-f*_ and *t*_*m-m*_), whereas reactive events follow events of the partner (*t*_*m-f*_ and *t*_*f-m*_, Fig. 2a). An interaction can be characterized by a transition between self-contained and reactive callings in male and female zebra finches (Fig. 2b).To determine the burst dynamics of calling interactions, we measured the inter-event intervals (*τ*), including the female self-contained inter-event intervals (*τ*_*f-f*_ = *t*_*f* i+1_ – *t*_*f* i_), the male self-contained inter-event intervals (*τ*_*m-m*_ = *t*_*m* j+1_ – *t*_*m* j_), the female reactive inter-event intervals (*τ*_*m-f*_ = *t*_*f* i_ – *t*_*m* j_) and the male reactive inter-event intervals (*τ*_*f-m*_ = *t*_*m* j_ – *t*_*f* i_) of the 22 pairs of zebra finches. By plotting the distribution of inter-event intervals, we found that the empirical cumulative distribution function (eCDF) of the self-contained inter-event intervals (i.e. *τ*_*f-f*_ and *τ*_*m-m*_) and the reactive inter-event intervals (*τ*_*m-f*_ and *τ*_*f-m*_) is approximated by a power-law distribution:

2 $${P}_{Power-Law}(\tau )={(\frac{\tau }{{\tau }_{\min }})}^{1-\alpha }$$

where *α* indicates the exponent and *τ*_*min*_ the lower bound (Fig. 2c, the example is taken from the female - male pair “a”, see Supplementary Fig. S1).

The power-law scaling is not valid for the full range of data. Lower value saturations (*τ*_*min*_) and higher value cut offs (*τ*_*max*_) are often observed in real data. In the mathematics of power-law, values of *τ*_*max*_ can go to infinity and the values of *τ*_*min*_ must be larger than 0. For this reason, there would be very large numbers of possible models fitting to the empirical data if all the lower values were considered for the fitting, i.e., there must be a lower bound value of *τ*_*min*_ below which the power-law does not make sense. The integral of power-law function *τ* ^-*α*^ over a certain range of values (τ) will converge only if the range is between *τ*_*min*_ and +∞, whereas the integral of power-law function will diverge when the values (τ) are approaching 0. Taken together, we fitted our data over a range from *τ*_*min*_ to the largest observed values. In contrast to power-law functions, the exponential functions are not constrained by a dynamic range.

The exponential cumulative distribution functions (Exp CDF):

3 $${P}_{Exp}(\tau )={e}^{\frac{-\tau }{\mu }}$$

with the corresponding measured average inter-event intervals (*μ*_*τ*_) did not capture the distribution of the data (Fig. 2c). Furthermore, the measured standard deviations of inter-event intervals (*σ*_*τ*_) were significantly larger than the estimated values (Tables 1 and Supplementary Table S1). In order to quantify statistical significance between the empirical data and the models, we fitted the data to the power-law cumulative distribution function (equation (2)) by using the fitting algorithms^32^. Log-log plots of the eCDF of *τ* display a heavy tail decreasing in the fashion of a straight line (Fig. 2c). The Exp CDF decreased much faster than the eCDF and the Kolmogorov-Smirnov test between the Power-law CDFs and the Exp CDFs rejected the hypothesis of exponential distribution (p < 0.001). In contrast, the Power-Law CDF fitted well to the data (p_*f-f*_ = 0.065, p_*m-m*_ = 0.605, p_*m-f*_ = 0.490, p_*f-m*_ = 0.029, Kolmogorov-Smirnov test). 83% of our data (73 of 88 modes of callings, i.e. 4 modes in each pair) could be fitted significantly to the power-law distribution (p > 0.01, Supplementary Fig. S1 and Supplementary Table S1). Data that failed fitting to the power-law distribution also failed fitting to the exponential distribution. Taken together, our analysis showed that the dynamics of calling interactions were described by power-law distributions with 2 < *α* < 3 (Table 1) in which the mean value of calling activity is finite, whereas the variance is divergent. A power–law function has a well-defined mean only if the exponent *α* > 2 and has a finite variance only if *α* > 3 (the mathematical explanations and further examples can be found in Supplementary Text S2 and Supplementary Fig. S4).

**Table 1:** Measured values and estimated parameters of the calling behavior.

	min *τ*	max *τ*	*μ*_*τ*_	*σ*_*τ*_	*τ*_*min*_	α
*f-f*	0.120 (0.100)	879.5 (197.6)	4.045 (2.438)	21.624 (11.718)	3.290 ± 3.270	2.34 ± 0.35
*m-m*	0.100 (0.002)	603.0 (526.7)	4.535 (2.989)	30.652 (17.992)	2.389 ± 1.148	2.24 ± 0.25
*m-f*	0 (0)	371.9 (160.9)	3.331 (1.436)	14.474 (7.384)	2.202 ± 1.625	2.41 ± 0.36
*f-m*	0 (0)	338.0 (191.9)	2.175 (1.501)	11.100 (6.786)	2.070 ± 1.103	2.42 ± 0.31

Abbreviations: *f-f*: female self-contained callings; *m-m*: male self-contained callings; *m-f*: female reactive callings; *f-m*: male reactive callings; min *τ*: minimum inter-event interval (in seconds); max *τ*: maximum inter-event interval (in seconds); *μ*_*τ*_: mean inter-event interval (in seconds); *σ*_*τ*_: standard deviation of inter-event interval (in seconds); *τ*_*min*_: lower bound for fitting algorithms (in seconds); *α*: exponent of power-law distribution. (measured value: median (25% quantile); estimated parameter: mean ± SD, n = 22).

Previous analyses of human activities suggested that power-law dynamics were a simple consequence of circadian cycles and Poisson processes^39, 40^, partially because the data included long periods of inactivity such as sleep during the night. To rule out this possibility, we excluded the nightly period of inactivity and sorted the calling events with respect to their inter-call intervals (*τ*) in a descending order, and then plotted the sorted calling events against the day-times. Neither the occurrences of calling events after short inter-call intervals nor the occurrences of calling events after long intervals had a circadian bias (Supplementary Fig. S5).

### The power-law scaling changes transiently in response to perturbations

**Figure 3:**
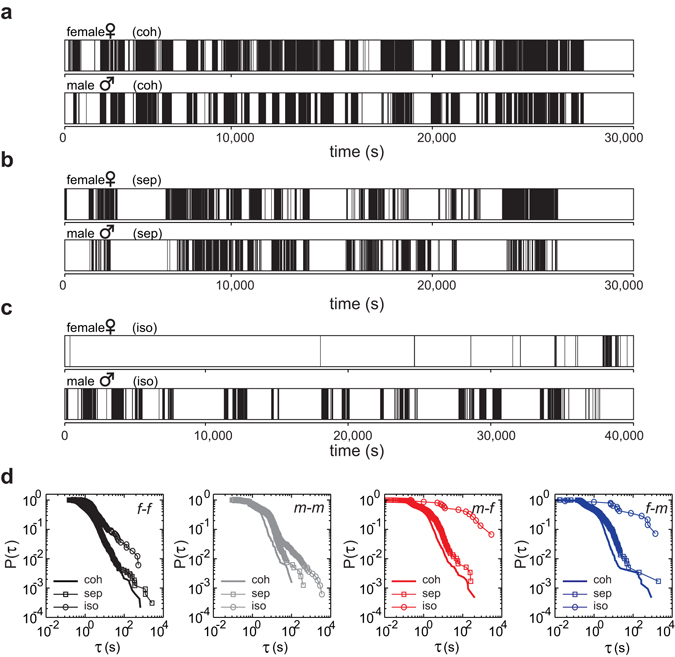
The power-law scaling of the self-contained (“*f-f*”, “*m-m*”) and of the reactive (“*m-f*”, “*f-m*”) callings change independently. Data are shown from the female-male pair “u”. (**a**) Successions of calling events of a female and a male during cohabitation (coh). (**b**) Successions of calling events of a female and a male after separation (sep) of the birds into two acoustically interconnected chambers allowing only acoustic interactions. (**c**) Successions of calling events of a female and a male after separating the birds into two chambers without any interconnection, neither visual nor auditory (iso = isolation). (**d**) Comparison of empirical cumulative distribution functions (eCDFs) between the inter-event intervals (*τ*) of cohabitation (coh), separation (sep), (sep) and isolation (iso). Note that the exponents changed dramatically only after isolation of the male and female zebra finches. P(*τ*): the cumulative distribution of the inter-event intervals; *τ* (s): the inter-event intervals in seconds.

First, we consider that the burstiness of inter-event intervals could be the consequence of a fixed behavioral pattern. Especially the reactive events might not be causally related to answering mate’s calls. Second, although we define the states of events based on the observation of calls, some calls might follow other activity, e.g. nonverbal gestures. Thus, it is possible that 1) the reactive dynamics could be the consequence of a self-contained behavior of each individual instead of one depending on external cues such as the partner’s calls, and that 2) the predefined reactive state is irrelevant for a reaction. In order to rule out these possibilities, we performed a sensory perturbation experiment by allowing zebra finch mates to interact only *via* microphones and loudspeakers. The results from one such pair are shown in Fig. 3: The power-law dynamics persisted during separation in which the reproductive partners could communicate acoustically (Fig. 3b and d, “sep”) and showed a similar mathematical function to that during cohabitation (Fig. 3a and d, “coh”) in all four modes of callings. Moreover, we measured the calling behavior during isolation (Fig. 3c and d, “iso”) in which the reproductive partners could not communicate acoustically or visually. After isolation, the exponents of the self-contained callings stayed at around 2 (Fig. 3d), whereas the exponents of the “reactive” callings decreased strongly below 0.5 during isolation (Fig. 3d, Supplementary Fig. S6). Since these “reactive” callings should be accidental, the numbers of “reactive” callings (m-f and f-m) are small (below or around 100). According to Clauset *et al*., the larger the sample size is, the more accurate is the estimated exponent. For small data sets, estimation error can be significant but should be smaller than the statistical error. A reliable and a good estimation can be achieved by providing more than 50 and 100 events, respectively^32^. Although we are not able delineate the mechanism how the isolation experiments impact on the self-contained callings in this study, we conclude that the calling activity of zebra finches follow power-low with exponents between 2 and 3. The power-law scaling of the calling dynamics revealed that the self-contained calling behavior is distinct from the reactive calling behavior.

**Figure 4:**
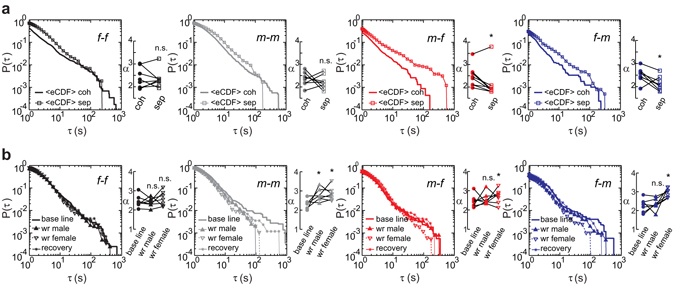
The exponents of different callings changed transiently in response to the social context (**a**) and the access to water (**b**). (**a**) Comparison of median empirical cumulative distribution functions (<eCDF>) between the inter-event intervals (*τ*) of “stack” calls during cohabitation (coh) and separation (sep). After limiting the social input to the auditory cues, the exponents (*α*) of reactive female (“*m-f*”) callings (p_*m-f*_ = 0.039, n = 8) and reactive male (“*f-m*”) callings (p_*f-m*_ = 0.027, n = 8) decreased while this did not change the exponents of self-contained female (“*f-f*”) and male (“*m-m*”) callings (p > 0.7, n = 8). (**b**) Comparison of <eCDFs> between the situations before, during and after removal of water. Removing water from males (wr male) increased the exponents (*α*) of self-contained male (“*m-m*”) callings (p_*m-m*_ = 0.023, n = 7). Removing water from females (wr female) increased the exponents of self-contained male (“*m-m*”) callings (p_*m-m*_ = 0.016, n = 7), of reactive female (“*m-f*”) callings (p_*m-f*_ = 0.016, n = 7) and of reactive male (“*f-m*”) callings (p_*f-m*_ = 0.008, n = 7). The other modes of callings remained unchanged (p > 0.050, n = 7). The exponents of base line and recovery were similar for all four modes of callings (p > 0.100, n = 7, recovery data are not shown on the ladder plots). n.s. and * indicate non-significant and significant, respectively. P(*τ*): the cumulative distribution of the inter-event intervals; *τ* (s): the inter-event intervals in seconds.

Subsequently, we tested the power-law scaling in response to biological relevant perturbations (1. reproductive partner is out of sight but in hearing range; 2. water is transiently not available) in eight zebra finch pairs (Fig. 4). The heavy-tailed dynamics were maintained unchanged during separation with acoustical interconnection (<eCDF> sep) as compared to that during cohabitation (<eCDF> coh) in all four modes of callings. The exponents of the self-contained female (p_*f-f*_ = 0.578) and male (p_*m-m*_ = 0.371) callings persisted unchanged as indicated by a two-sided Wilcoxon signed-rank test (n = 8), whereas the exponents of the reactive female (p_*m-f*_ = 0.027) and male (p_*f-m*_ = 0.039) callings decreased during separation with acoustical interconnection as indicated by a right-sided Wilcoxon signed-rank test (Fig. 4a, n = 8). This indicates that the self-contained callings are indeed self-contained and not affected by the social-sensory environment, whereas the reactive callings depend on multiple-sensory cues. However, we could not exclude the possibility that some reactive callings were by chance. Such incidental “reactive” callings can be considered as an experimental noise, as any other experimental noise. Nevertheless, the perturbation experiments suggest that the incidental noise is not the main factor in this system.

To probe the plasticity of the self-contained callings, we performed a water-removal experiment with seven pairs. Water-removal did not change the overall power-law distribution of calling intervals. Water-removal from males resulted in a significant (left-sided Wilcoxon signed-rank test, n = 7) increase of the exponents of the self-contained male callings (p_*m-m*_ = 0.023, Fig. 4b). The exponents of the self-contained female callings, the reactive female and reactive male callings remained unchanged by water-removal from males (Fig. 4b, p > 0.050, two-sided Wilcoxon signed-rank test, n = 7). As indicated by left-sided Wilcoxon signed-rank tests (n = 7), water-removal from females not only increased the exponents of the self-contained male callings (p_*m-m*_ = 0.016) but also increased the exponents of the reactive female (p_*m-f*_ = 0.016) and reactive male (p_*f-m*_ = 0.008) callings (Fig. 4b). Neither water-removal from males nor from females had an effect on the exponents of the self-contained female callings (Fig. 4b). Although the exponents of self-contained female callings had not changed, the distributions of call rates in both females and males changed in response to water-removal. The call rates became more irregular in male compared with females (Supplementary Fig. S2, Supplementary Text S1). Finally, the exponents returned to their initial values after the end of the treatments (Fig. 4b), i.e. the exponent (signature) of both reactive and self-contained callings of a particular zebra finch is homeostatically regulated over at least 10 days.

## Discussion

Zebra finches, like many other group living vertebrates produce large numbers of sounds per day^1, 3, 10, 41^. Calls that are answered by others or calls with a predetermined meaning, e.g. advertising calls, are generally thought to be biologically meaningful^1, 5, 8, 10^. However, calls without identified meanings that are not answered by conspecifics are a mystery. Next to answered “stack” calls, zebra finches of both sexes utter thousands of un-answered “stack” calls that are acoustically identical to the answered ones^17^. We show that the time intervals of both the self-contained “stack” callings and the reactive (answered and answers) “stack” callings of zebra finch pairs are characterized by a power-law distribution. This power-law model provides an accurate statistical description of the calling activity that changed transiently with varying physiological states. In particular, the calling activity during separation and water-removal experiments remained power-law distributed with exponents ranging between 2 and 3, indicating that the zebra finches maintained calling rates within bounds during altered environmental conditions and recovered to pre-perturbation values after the birds were re-supplied with water. Although the power-law distribution was frequently suggested for large data sets^31, 32, 42^, there are actually very few good biological examples^33^, including allometric scaling of metabolic rate^43^ and power-law distribution of biological taxa^44^. The present study reports the first power-law scaling of vocal communications of vertebrates and extends the analysis of vocal time series that were previously limited to short time ranges of vocal contingency^17, 18^. Next to the impact of social interactions^22, 23^, calling dynamics were regulated by the physiological states in zebra finches.

How could the power-law properties be biologically informative? Due to the scale-free properties of the power-law behavior, a receiver such as the mate may just need to analyze a fraction of all inter-call intervals to compute the expected distribution of daily calling intervals. Since this distribution is stable under normal conditions, a conspecific could learn the activity pattern of a focal individual, e.g. of their reproductive partner. The actual signature (exponent) of the calling pattern of an individual would inform the pre-informed receiver about deviations that reflect social or physiological disturbances of the calling individual. In relation, since the acoustic structure of “stack” calls is almost invariant^10, 45^, the sequential hearing of several “stack” calls contains very limited information. However, information could be encoded in varying the inter-call intervals, which is reflected in the exponent of the power-law function. Adjusting the exponent of the callings may extend the information capacity limited by invariant calls such as the “stack” calls.

The power-law characteristics of “stack” calls allowed us to study the biological dynamics of this behavior. We tested its stability under different environmental conditions: (1) visual separation of the reproductive partner, (2) auditory and visual separation of the reproductive partners (isolation), and (3) temporarily restricted access to water (see Materials and Methods)^23, 34^. We chose these conditions to simulate important situations in the life of zebra finches assuming that vocal communication of captive zebra finches is similar to their wild-living conspecifics. These birds live in the grasslands of Australia, mate for life and breed opportunistically following sporadic rainfall^46^. Vocal contact with the mate during foraging or dispersal in grassland is certainly part of their pair-maintenance behavior. Rainfall is essential to initiate breeding periods although the animals are well adapted to survive without drinking water for very long periods^34^. Each of the simulated conditions affected the exponent of the power-law function of either the self-contained calling, the reactive calling, or of both. The direction of change differed between experiments. A decrease of the exponent was observed following isolation and an increase of the exponent was observed following water removal. Furthermore, the distribution of the call intervals of the visually deprived or water-restricted males and females relaxed to the pre-perturbation values once the situation normalized. Thus, the daily calling intervals (ranging from 50 milliseconds to 20 minutes) of mated male and female zebra finches are characterized by individual signatures that are stable over days and that are sensitive to varying sensory and physiological states. This long-term stability of calling pattern may indicate an important role for pair maintenance and success. Another role of the power-law behavior of calling activity might concern the situations in which the mate is out of visual and auditory detection range^23^. To know that long call intervals occur regularly might prevent zebra finches immediately searching for their reproductive partner once it is out of sight. Because zebra finches mate for life and are opportunistic breeders, information about the mate and therefore well-synchronized vocal behaviors could be important for breeding success^18^, as suggested based on the temporal correlations of reactive callings.

Some data that are approximated by a power-law distribution can also be modeled by the lognormal distribution (e.g. Gibrat’s proportionate growth model)^47^. These are feasible models for natural phenomena and have comparable distribution properties, which result in very similar regressions over a range of inter-event interval values (*τ*_*i*_) between *τ*_*min*_ and *τ*_*max*_^47^. Gibrat’s law may also hold for our data when assuming a growth process^47–49^, where two zebra finches start to communicate with an initial set of *τ*_0_. At each step, they may accelerate or reduce the rate of response times. After sufficient steps, their inter-event intervals (*τ*) change proportionately, independent of initial *τ*_0_ and would be lognormally distributed. Besides and lognormal models, it is plausible that Weibull^50^, bimodal distributions^51^, and time-scale segmenting^30^ may capture the dynamic patterns of daily calling interactions. However, all these models such as Gibrat’s law explain only 50% of our data sets (44 of 88 modes of callings based on 22 pairs and 4 modes of calling per pair) while the power-law distribution explains 83% of our data sets. Our statistical tests and empirical measurements (Table 1, Fig. 1, Supplementary Table S1) showed that the daily moment-by-moment calling interactions of zebra finches, consisting of self-contained and reactive calling activity are best approximated by a power-law model.

Our data suggest two types of calling behavior in zebra finches: the self-contained and the reactive calling. This distinction is supported by the independent changes of the signatures of these two behaviors, e.g. after separation of the mates only the reactive calling behavior changes. It remains, however, to be seen if self-contained callings and reactive callings are controlled by separate vocal circuits, as suggested for primates^52^, and how internal states could influence the homeostasis of calling behavior. Nevertheless, our study suggests that the utterance of large numbers of calls as seen in many birds^1, 10, 14^ and mammals^3, 11, 41, 53^ is a characterization of the physiological state of the sender, even though a single call has no predetermined meaning.

## Materials and Methods

### Animals

Animal care and experiments were approved by the Government of Upper Bavaria (Az. 55.2-1-54-231-25-09). All further animal husbandry or handling was conducted according to the directives 2010/63/EU of the European parliament and of the council of 22 September 2010 on the protection of animals used for scientific purposes. Experimental animals were adult male and female zebra finches obtained from our breeding facility. Zebra finches were kept in a 14/10 (Light/Dark) cycle and provided with food and water *ad libitum*. For pair-formation, one female and one male bird were force-paired by keeping them in cages (size: 54x40x28 cm), placed in custom-made, sound-attenuated boxes. Each sound box was equipped with a microphone (C2, Behringer, Willich-Münchheide II, Germany), a speaker (FRS 8, 30w, 8Ω, VISATON, Germany) and a telescopic antenna for wireless recordings. 22 male-female pairs of zebra finches (44 birds) were tested for the power-law scaling. 8 of these 22 pairs were used for the separation experiments. In addition, 7 pairs were used for the water-removal experiments and 3 pairs were used for the isolation experiments.

### Sound recording

Zebra finches were kept under a light/dark cycle of 7:00/21:00 that corresponds to the active/inactive cycle. The vocalizations of zebra finches were recorded for 4 to 18 hours per light/dark cycle (Supplementary Table S1). Even though zebra finches are usually inactive during the dark cycle, the vocalizations of both male and female birds were recorded continuously during the recording periods, such that the data showed the precise daily on/off vocal activity. Each bird was equipped with a custom-made wireless microphone transmitters (0.6 g including a battery)^17^. The microphone was placed on the back and fixed with an elastic band around the upper thighs of the bird. The frequency modulated radio signals were received with communication receivers (AOR5000, AOR Ltd., Japan). Audio signals were either fed into an eight channel audio A/D converter (Fast Track Ultra 8R, Avid Technology, Inc. U.S.A.) and recorded with custom-made software or registered on a data recorder (DASH8X, Astro-Med Inc., RI, U.S.A.). The audio signals were recorded continuously and written to WAV files. Vocalizations were extracted from audio files by using custom-written software (sound explorer, available at https://github.com/ornith). The vocalizations of zebra finches were clustered by analyzing their sound features^17^.

### Perturbation experiments

1. Separation: Eight zebra finch pairs were used for separation by limiting visual cues emerging from their mates (ID: c, i, j, k, l, m, n, o corresponding to Supplementary Table S1). Male and female zebra finches were separated into 2 sound-attenuated boxes for four hours. Animals were communicating via wired microphones and speakers, which delayed the communication by less than 1 millisecond (ms) (Supplementary Fig. S3).
2. Isolation: The calling activity of the male and female partner was tested in isolation (visual and acoustic separation) by separating the partners into 2 sound-boxes without acoustic and visual interconnections for eleven hours (N = 3 pairs).
3. Temporarily restricted access to water: Seven zebra finch pairs (ID: p – v) were used for the water-removal experiment. Each pair was first put into a recording chamber for cohabitation during 5–7 days prior to the treatment. The procedure of the treatment was as follows: 1 day of water-removal from the male; 2–3 days of recovery; 1 day of water-removal from the female; 2–3 days of recovery. Vocalizations of both male and female birds were recorded continuously through backpack microphones. During the water-removal experiments, mates were separated with a transparent plastic board, so that the male and the female had auditory and visual contact, but only one mate had access to the water. Food was provided *ad libitum* for both males and females during the procedure.

### Data analysis

The periodogram S(*f*) estimates the contributions of each frequency (*f*) to the signal. Let us consider the change of call rates (Δγ) in time (t). We obtained a time series Δγ_1_, Δγ_2_, …, Δγ_i_ at times t_1_, t_2_, …, t_i_. The periodogram S_Δγ_(*f*) of a time series Δγ(t) is defined as the contribution of each frequency *f* to the time series Δγ(t). An uncorrelated white noise will appear as a flat line (i.e. slope ~ 0) on the periodogram. If a time series has a high power at low frequency, this time series has a long-range fluctuation. By contrast, if a time series has a high power at high frequency, this time series has a short-range fluctuation.

The fitting procedure^32, 54^ aimed to estimate the exponent from a discrete set of inter-event intervals from a lower bound: *τ*_*min*_ to any possible largest value in a continuous recording (about 1,000 seconds for our longest observation period: 10 hours during the Light cycle). The steps of the fitting procedure in our study were as follows:

1. Let the inter-event intervals be *τ*_*i*_, *i* = 1, …, n. We estimated the candidate exponents *α*′ and the candidate *τ*′_*min*_ between the shortest and the longest inter-call intervals. The larger *n* we have, the more precise the estimated *α*′ is approximated by the true *α* ^32^.
  4 $$\alpha ^{\prime} =1+n{[\sum _{i=1}^{n}\mathrm{ln}\frac{{\tau }_{i}}{{\tau }_{\min }^{^{\prime} }-\frac{1}{2}}]}^{-1}$$
2. Many (*α*′, *τ*′_*min*_) pairs will be obtained from step 1. Assuming that the discrete power-law distribution is:
  5 $$p(\tau )=\frac{1}{\zeta (\alpha ,{\tau }_{\min })}{\tau }^{-\alpha }$$
  thus the cumulative distribution function (CDF) will be:
  6 $$p^{\prime} (\tau )=1-\frac{\zeta (\alpha ,\tau )}{\zeta (\alpha ,{\tau }_{{\rm{\min }}})}$$
3. Calculate the distance (D) between the empirical CDF and the estimated CDF:
  7 $$D={\max }_{\tau \ge {\tau }_{\min }}|eCDF(\tau )-{P}^{^{\prime} }(\tau )|$$
4. Repeat steps 1–3 until we have all possible (*α*′, *τ*′_*min*_) pairs calculated by whole range of *τ*′_*min*_ from the shortest (i.e. min *τ*) and the longest inter-event intervals (i.e. max *τ*). By looking at the plot of distance (D) against *τ*′_*min*_, we obtain the minimum distance with corresponding *τ*_*min*_ and exponent *α*.
5. Calculate the goodness of the fit of the exponential and lognormal distributions to the real data. The Kolmogorov-Smirnov statistic was used to quantify the distance between the empirical CDF and the synthetic CDF with corresponding parameters. The closer the p-value is to 1, the more the simulated data with corresponding *α* and *τ*_*min*_ are similar to the real data.

Maximum likelihood estimation (mle, in MATLAB, Mathworks) was used to estimate the parameters (*θ*) of the exponential model (*μ*) and the lognormal model (*μ*, *σ*). Let the inter-event intervals be *τ*_*i*_, *i* = 1, …, n. The parameters are estimated by maximizing the log-likelihood function:

8 $$\mathrm{log}\,L(\theta ;{\tau }_{1},{\tau }_{2},\ldots ,{\tau }_{i})=\sum _{i=1}^{n}\mathrm{log}({\tau }_{i}|\theta ),$$

where $p({\tau }_{n}|\theta )$ is the likelihood probability function. The parameters *θ* can be one parameter *(μ*) for exponential function and multiple parameters *(μ*, *σ*) for the lognormal function. All data points (i.e. form any possible smallest to any possible largest value in a continuous recording) were used for the maximum likelihood estimation. To estimate the goodness of the fits of exponential and lognormal distributions to the data, we used Kolmogorov-Smirnov statistic to test the distance between the empirical CDF and the estimated CDF.

The empirical cumulative distribution function (eCDF) is shown by a plot of events against the sorted inter-event intervals (*τ*) with corresponding indices. We used the One-sample Kolmogorov-Smirnov test to test whether the empirical data follow the exponential distribution. The Power-law distribution was estimated using fitting algorithms as described by Clauset *et al*.^32^. The decreases of exponents in all variables were analyzed with a right-sided Wilcoxon signed-rank test by testing whether the data (e.g. *α*_*cohabitation*_ – *α*_*separation*_) came from a distribution with a median greater than 0. The increase of exponents was analyzed with a left-sided Wilcoxon signed-rank test by testing whether the data (e.g. *α*_*base line*_ – *α*_*wr male*_) originated from a distribution with a median smaller than 0. The two-sided Wilcoxon signed-rank test was used to analyze whether the data (e.g. *α*_*cohabitation*_ – *α*_*separation*_) came from a distribution with a median different than 0. The test results are considered for significance at a 5% confidence level. The one sided Wilcoxon signed-rank test was performed only after the two-sided Wilcoxon signed-rank test indicated significance.

## Electronic supplementary material

Supplementary Information

Supplementary Table S1
