# Supplementary material for: Power-law scaling of calling dynamics in zebra finches
Source: Sci Rep. 2017 Aug 21;7:8397. doi: 10.1038/s41598-017-08389-w (PMC5566443; doi:10.1038/s41598-017-08389-w)
Supplement: Supplementary file 1 — Supplementary Information [file 41598_2017_8389_MOESM1_ESM.doc]

**Supplementary Information**

Power-law scaling of calling dynamics in zebra finches

Shouwen Ma^1,2^, Andries Ter Maat^1^, Manfred Gahr^1,2^

^1^ Max Planck Institute for Ornithology, Eberhard-Gwinner-Straße, 82319, Seewiesen, Germany.

^2^Graduate School of Systemic Neurosciences (GSN), Ludwig-Maximilians-Universität München, Großhaderner Str. 2, 82182 Martinsried, Germany.

**Supplementary Text S1**

**Quantitative analysis of calling activity in response to water-removal.**

We consider a system where the change of calling activity (ΔN) with respect to time (Δt) corresponds to the rate (γ). Thus we obtain: $\frac{\Delta N_{i}}{\Delta t_{i}}=\gamma_{i}$ (1), where, *i* indicates individual males or females. This equation accounted for a linear dynamics and the number of vocal events grew positively with time. Our measurements showed that temporary water-removal did not change the calling activity, neither the linear accumulation of calls over time (Supplementary Fig. S2b [i.] & 2c [i.]), nor the diversity of instantaneous call rates (Supplementary Fig. S2b [ii.] & S2c [ii.]), nor the changes of call rates (Supplementary Fig. S2b [iii], S2b [iv.], S2c [iii.] & S2c [iv.]). It also suggests that the temporary water-removal does not cause severe abnormality in calling behaviors. Finally, we analyzed the probability distribution of the call rates. We found that the treatment of water-removal affected the distributions of the call rates in both male and female, although just one individual was treated at a time. Before these treatments, the likelihood to call with lower rates was higher than the likelihood to call with higher rates in the female and the male (Supplementary Fig. S2a [v.]). During the water-removal from the male (Supplementary Fig. S2b [v.]) and from the female (Supplementary Fig. S2c [v.]), the likelihood to call with lower rates changed in the female whereas the call rates of the male became irregular and were distributed nearly equally between minimum and maximum call rates. The distribution of call rates in the male and in the female returned to a similar pattern ones the animals had again access to water (Supplementary Fig. S2d [v.]). Although we did not find a consistent pattern for the call rates, we assume that 1) regularity of call rates and 2) maintaining the changes of call rates (i.e. the acceleration) within bounds during calling interactions are two important factors for the scaling property of interaction dynamics. However, we were unable to derive an accurate mechanistic model (e.g. no power-law function) that would analytically predicts the call rates in vocal interactions.

**Supplementary Text S2**

**The moments and the periodicity of the power-law distribution**

We showed that the calling activity followed a power-law with exponents range between 2 and 3 in which the mean value of calling activity is finite, whereas the variance is divergent. According to the moment in mathematics, the nth moment of a distribution is defined as: $<\tau^{n}>\sim\int_{\tau}^{\infty} \tau^{n}p(\tau)d\tau$ (9), where the *τ* is the inter-event interval and the *p*(*τ*) is the probability function. The first moment: <*τ*> is the mean inter-event interval. The second moment: <*τ^2^*> is the variance of inter-event intervals. While there are a *τ_min_* and a *τ_max_* for a power-law distribution, the nth moment will be: $<\tau^{n}>=\frac{\tau_{\max}^{-\alpha+n+1}-\tau_{\min}^{-\alpha+n+1}}{-\alpha+n+1}$ (10). The value of <*τ^n^*> depends on $-\alpha+n+1$ ^54^. If n ≤ *α* – 1, the value of <*τ^n^*> will be finite as *τ_max_* goes to ∞. If n > *α* – 1, the value of <*τ^n^*> will be infinity as *τ_max_* goes to ∞. For empirical data, the *τ_max_* always exists. The measured variance of the data that follow power-law must be significantly larger than the measured mean values and the variance of the data that follow lognormal or exponential distributions. By contrast, the tail distribution of this “reactive” behaviors (Fig. 3c) have exponents much smaller than 2 (Supplementary Fig. S6, n = 3). As predicted by the moment <*τ^n^*>, neither the mean nor the variance will be finite. The measured mean (*μ*_f-m_ = 208.5 s and *μ*_m-f_ = 333.2 s) and variance (*σ*_f-m_ = 392.1 s and *σ*_m-f_ = 750.6 s) are large.

Cricket songs are considered as one of the fixed action patterns ^55^. We recorded two song bouts from a field cricket in isolation that contained 150 and 133 trills, respectively. Each trill contained 3 – 4 syllables. We measured the inter-trill intervals of these two song bouts. The exponents of these two song bouts are 4.01 (*μ*_1_ = 0.33 s, *σ*_1_ = 0.14 s, n_1_ = 150 trills) and 4.22 (*μ*_2_ = 0.38 s, *σ*_2_ = 0.18 s, n_2_ = 133 trills), respectively. The measured means and variances agree with the prediction of the moment in which n < α – 1, hence both mean and variance will be finite. Furthermore, the periodograms show that the trill rates (γ) of the cricket’ songs have a spectrum with higher power (S_γ_(f)) at lower frequencies (i.e. a long-range of fluctuation), and the changes of the trill rates (Δγ) have a spectrum with equal power (S_Δγ_(f)) at every frequency (i.e. slope ~ 0) (Supplementary Fig. S4). These analyses support a Brownian characteristic, i.e. a stochastic process in generating trills of the cricket’s songs. In contrast to the self-similar patterns (i.e. the normal calling activity of zebra finches) and the fixed action patterns (i.e. cricket’s song), the signals of uncorrelated behaviors should have equal density at every frequency as expressed by the periodograms of both γ and Δγ. As predicted, the “reactive” calling of the isolated zebra finch mates (*α* ≤ 2, depicted in Fig. 3d) exhibited an uncorrelated Δγ (i.e. slope ~ 0) and a week long-range of fluctuations in γ (i.e. the γ has a spectrum with small power in the low frequency that constitutes a large part of the signal,) suggesting an uncorrelated behavior (Supplementary Fig. S4).

**Supplementary Table S1. Estimated parameters and statistical tests for different zebra finch pairs during cohabitation before treatments** (The entire table S1 can be found as an Excel file online).

Abbreviations: **ID**: identities of the tested zebra finch pairs; **Period**: the start and the end of a recording; **Duration**: the duration of each recording in seconds (s); **Sex**: F: female, M: male; **Symbol**: f-f: female self-contained callings, m-m: male self-contained callings, m-f: female reactive callings, f-m: male reactive callings; **N**: number of events; **min *τ***: minimum inter-event interval (in seconds); **max *τ***: maximum inter-event interval (in seconds); ***μ****_τ_*: average inter-event interval (in seconds); ***σ****_τ_*: standard deviation of inter-event interval (in seconds); ***τ*_min_**: lower bound for fitting algorithms (in seconds); ***α***: exponent of power-law distribution; **p*_pl_***: p-value indicates the statistical significance of the fit to the power-law, the closer p-value is to 1, the more likely that the empirical data fit the distribution. The model accepts p > 0.01 for a significant fitting. ***μ_logN_***: indicates the mean value (location) of the data’s natural logarithm. ***σ_logN_***: indicates the standard deviation (scale) of the data’s natural logarithm. **p*_logN_***: p-value indicates the statistical significance of the fit to the lognormal distribution model, the closer p-value is to 1, the more likely that the empirical data fit to the model. The model accepts p > 0.01 for a significant fitting. N = 22 pairs.

**Supplementary Figures**

**Supplementary** **Figure S1**

**Supplementary** **Fig. S1a – S1o: Successions of calling interactions between females and males of all zebra finch pairs (pairs a – o) not exposed to any treatment.** Comparisons between empirical data and exponential cumulative density models with estimated parameters (Supplementary Table S1) for the self-contained female callings (**i.**), the self-contained male callings (**ii.**), the reactive female callings (**iii.**) and the reactive male callings (**iv.**). P(*τ*): the cumulative distribution of the inter-event intervals; *τ* (s): the inter-event intervals in seconds.

**
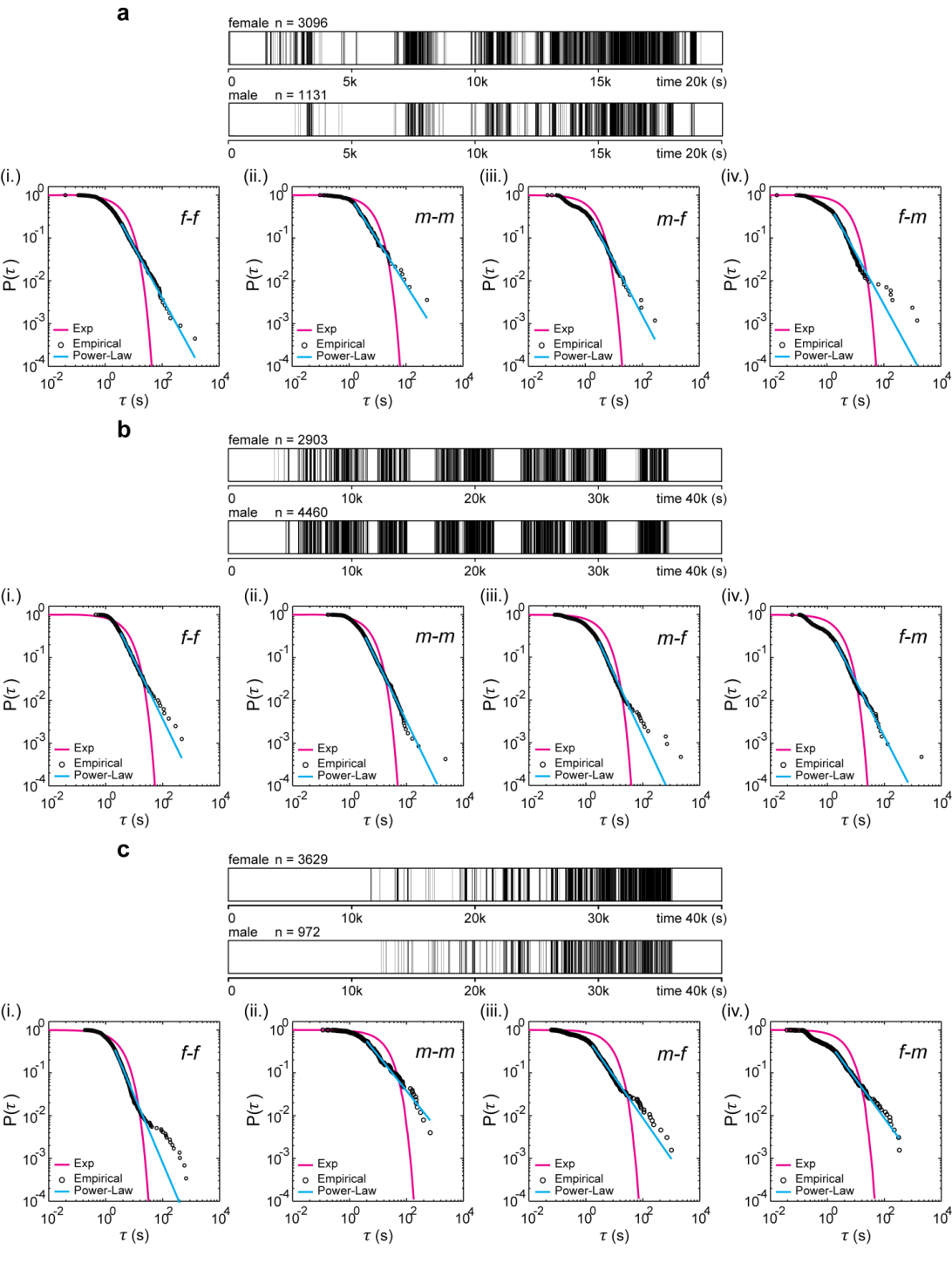

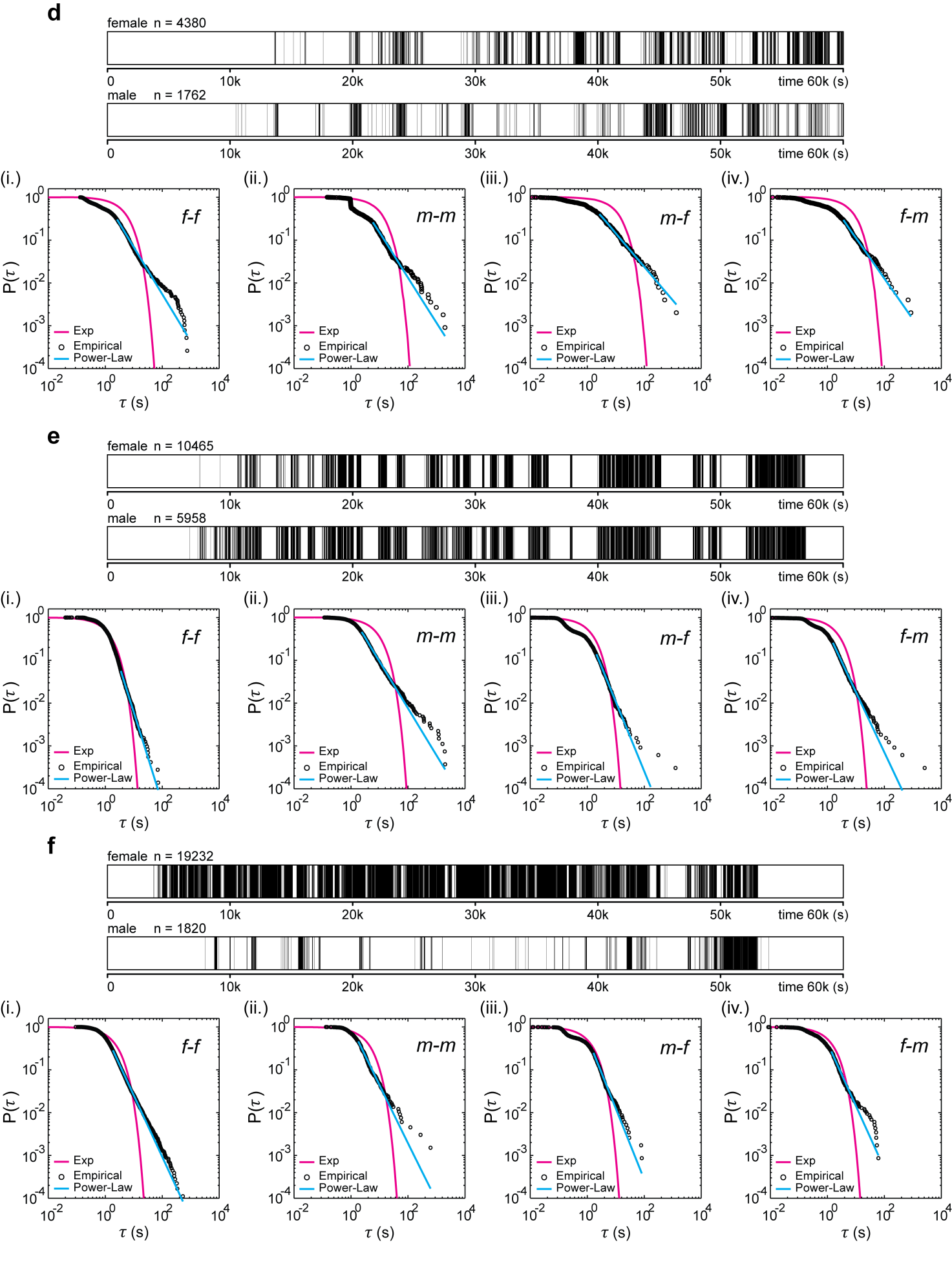
**

**
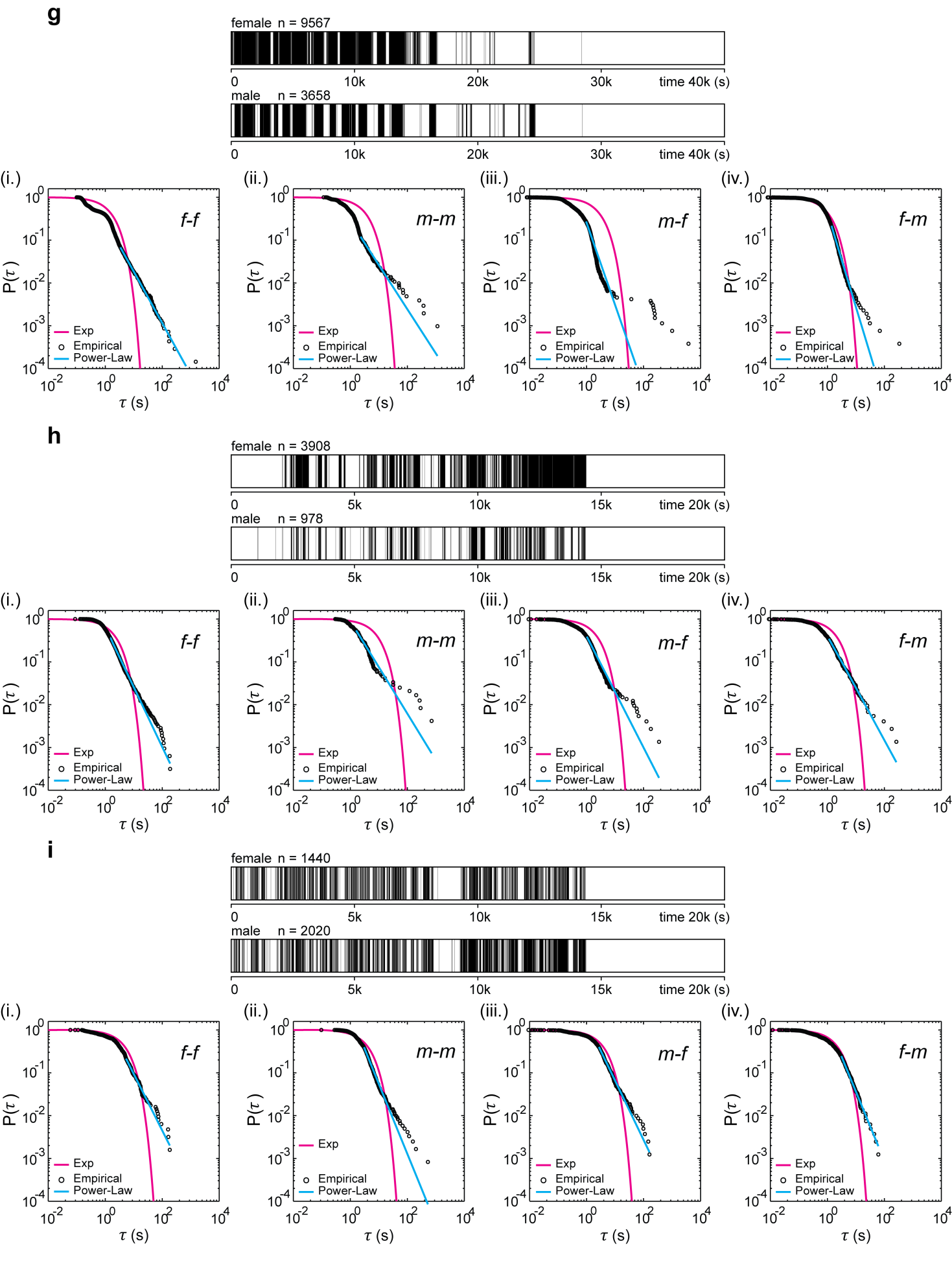
**

**
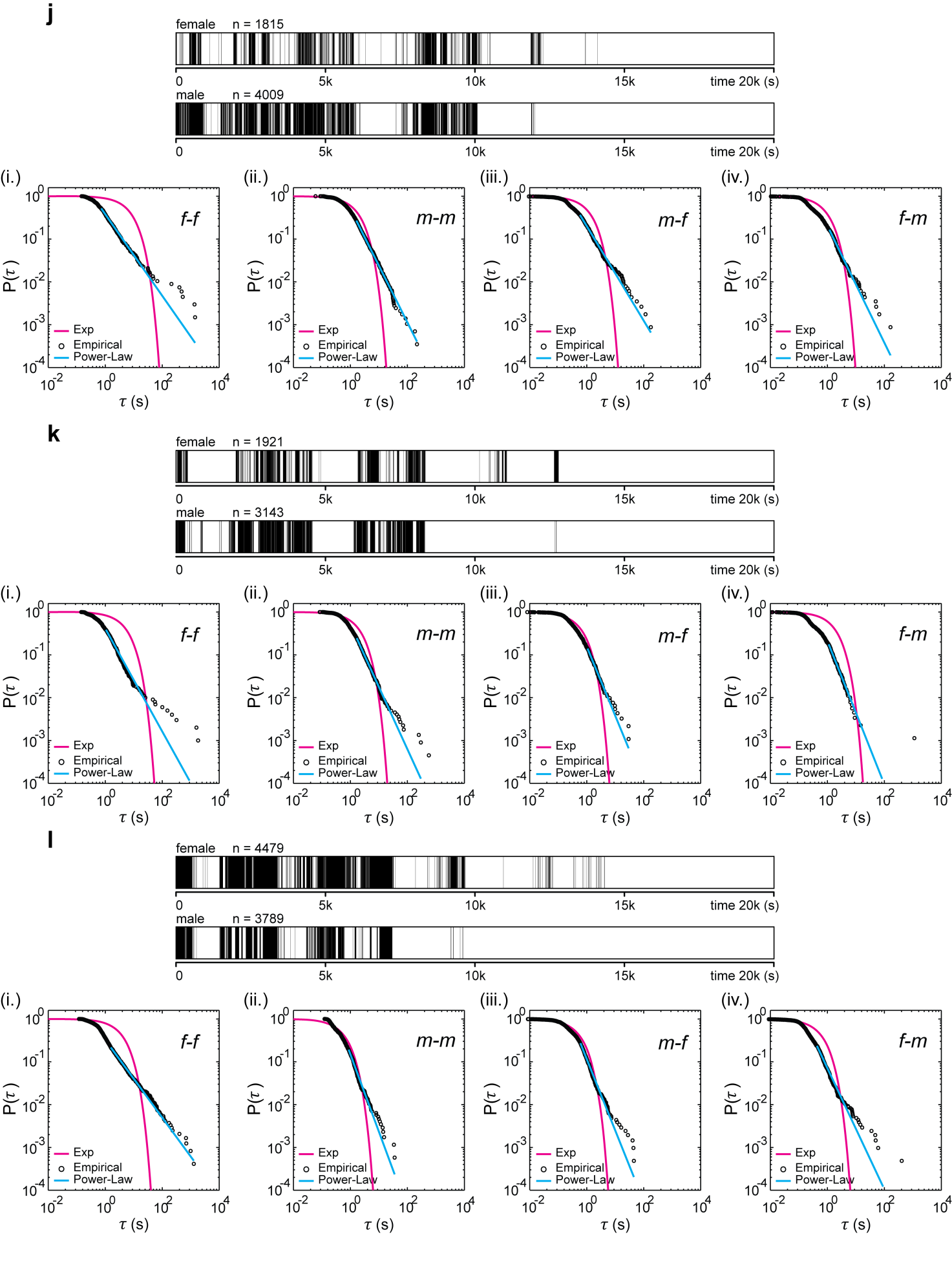
**

**
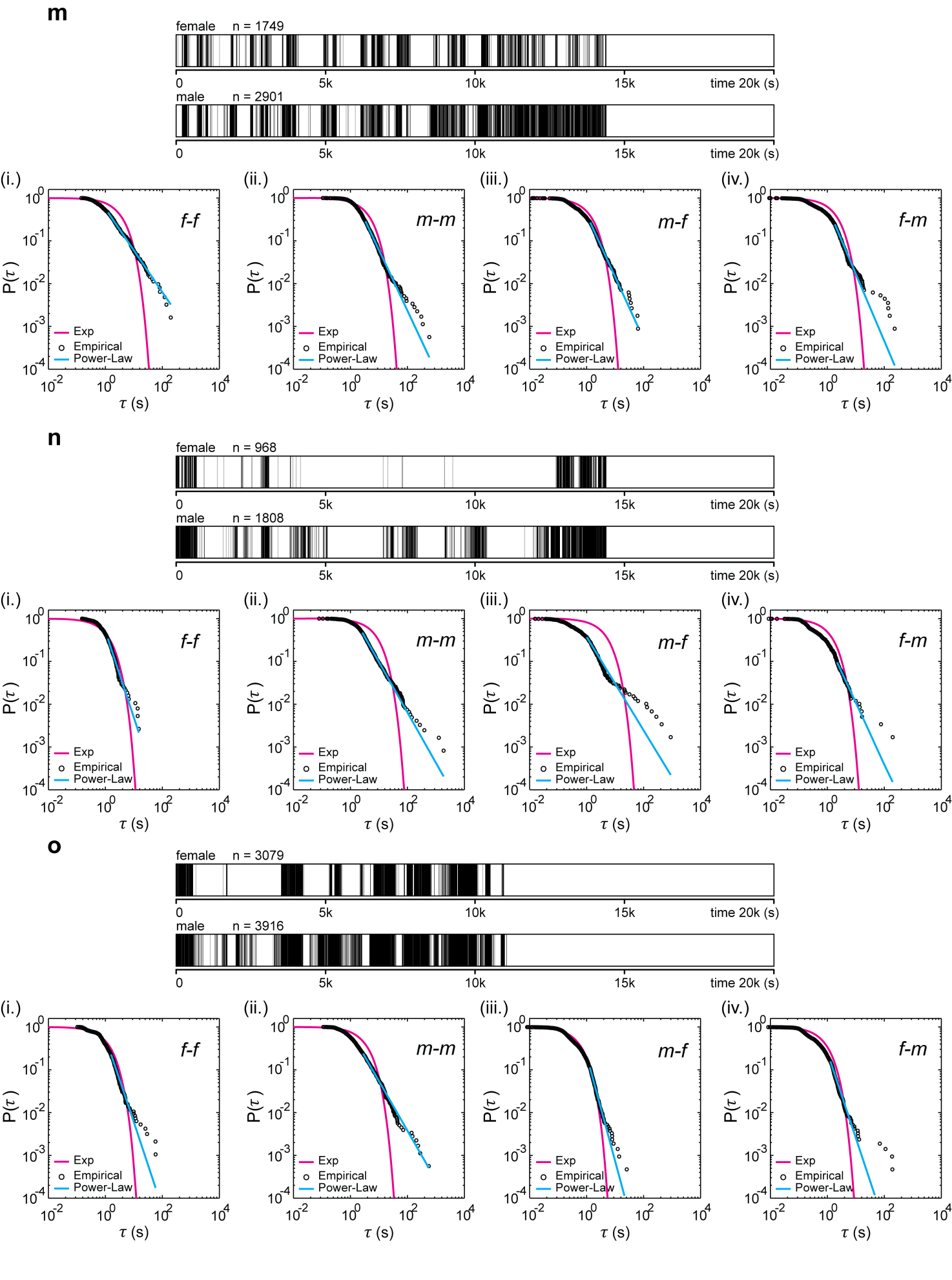
**

**Supplementary Figure S2**


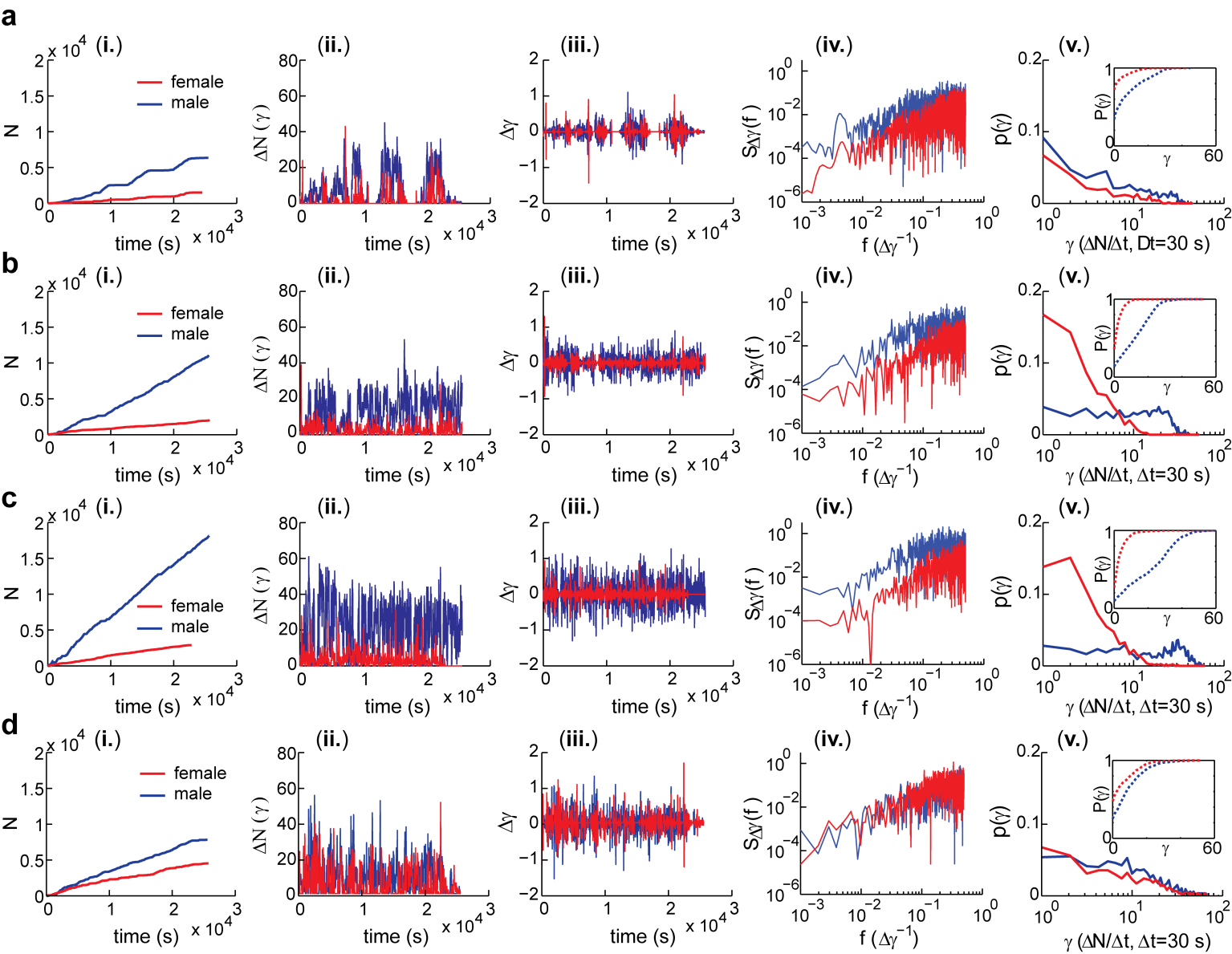


**Supplementary** **Fig. S2. Water-removal influences the distribution of the call rates of both male and female zebra finches.** Shown are four measurements, **(a)** providing water and food *ad libitum* for both sexes, (**b)** removing water only from the male, (**c)** removing water only from the female, and (**d**) providing water and food *ad libitum* for both mates after the end of experiment. In a-d, (**i.**): the cumulative number of calls over 7 – 8 hours of recording. (**ii.**): The call rates (γ) correspond to the number of calls (ΔN) in a unit of time (∆t = 30 sec); (**iii.**): The changes of call rates (∆γ); (**iv.**): The power spectrum of Δγ; (**v.**): The probability density functions of call rates (p(γ)). The inset displays the corresponding cumulative density function (P(γ)) of the treatments a – d, respectively.

**Supplementary Figure S3**

**
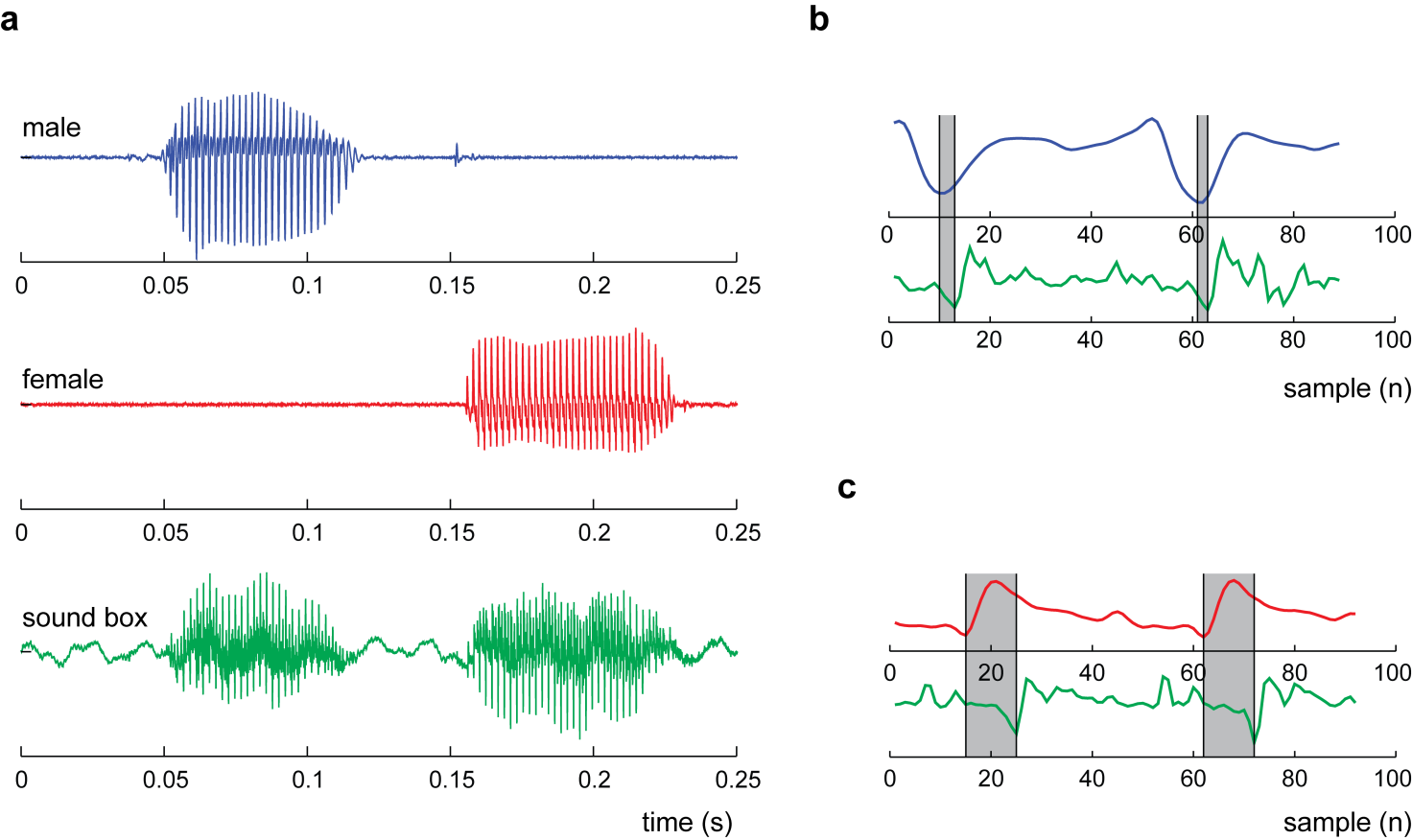
**

**Supplementary** **Fig. S3. Synchronous recording of vocal interactions during sensory perturbation.** (**a**) illustrates the segments of male (blue) and female (red) backpack recordings. Both male and female vocalizations were recorded by a microphone in the male-hosted sound box (green). **b**: By comparing the time shifts between the sound waves, we found a size of 3 samples (0.1 ms, sample rate: 22050) discrepancy between the male backpack recording and the male-hosted sound box. **c**: a size of 10 samples (0.5 ms, sample rate: 22050) discrepancy between the female backpack recording and the male-hosted sound box.

**Supplementary Figure S4**


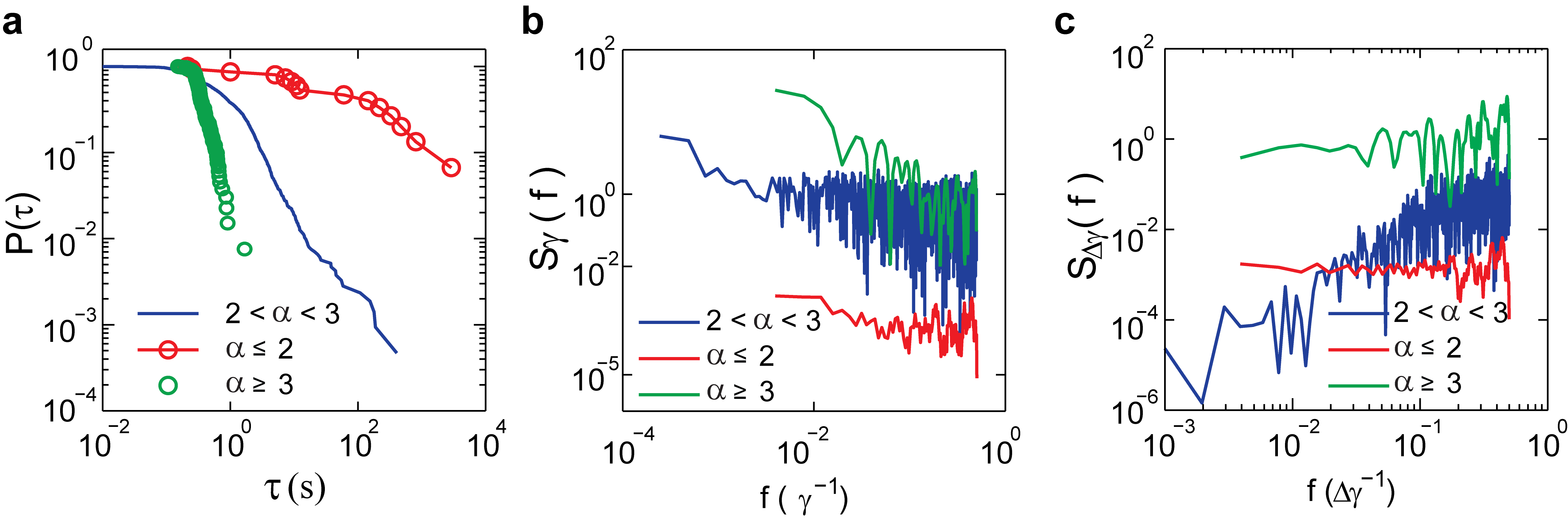


**Supplementary Fig. S4.** **Comparing the tail distributions of different behavioral categories.** (**a**) The tail distributions of inter-event intervals that fall into three different behavioral categories: (1.) “reactive” callings of isolated zebra finches (*α* ≤ 2), (2.) the natural calling activity of zebra finches (2 < *α* < 3), (3.) the trills of cricket songs (3 ≤ *α*). (**b**) The periodograms of the instantaneous rates (γ) of the three different behaviors depicted in (a). (**c**) The periodograms of the change of the instantaneous rates (Δγ) of three different behaviors depicted in (a). Note that the signal has a long-range fluctuation if the signal has a high power at low frequency (i.e. slope < 0). The signal has a short-range fluctuation if the signal has a high power at high frequency (i.e. slope > 0). The white noise signal has equal power at every frequency (i.e. slope ~ 0).

**Supplementary Figure S5**


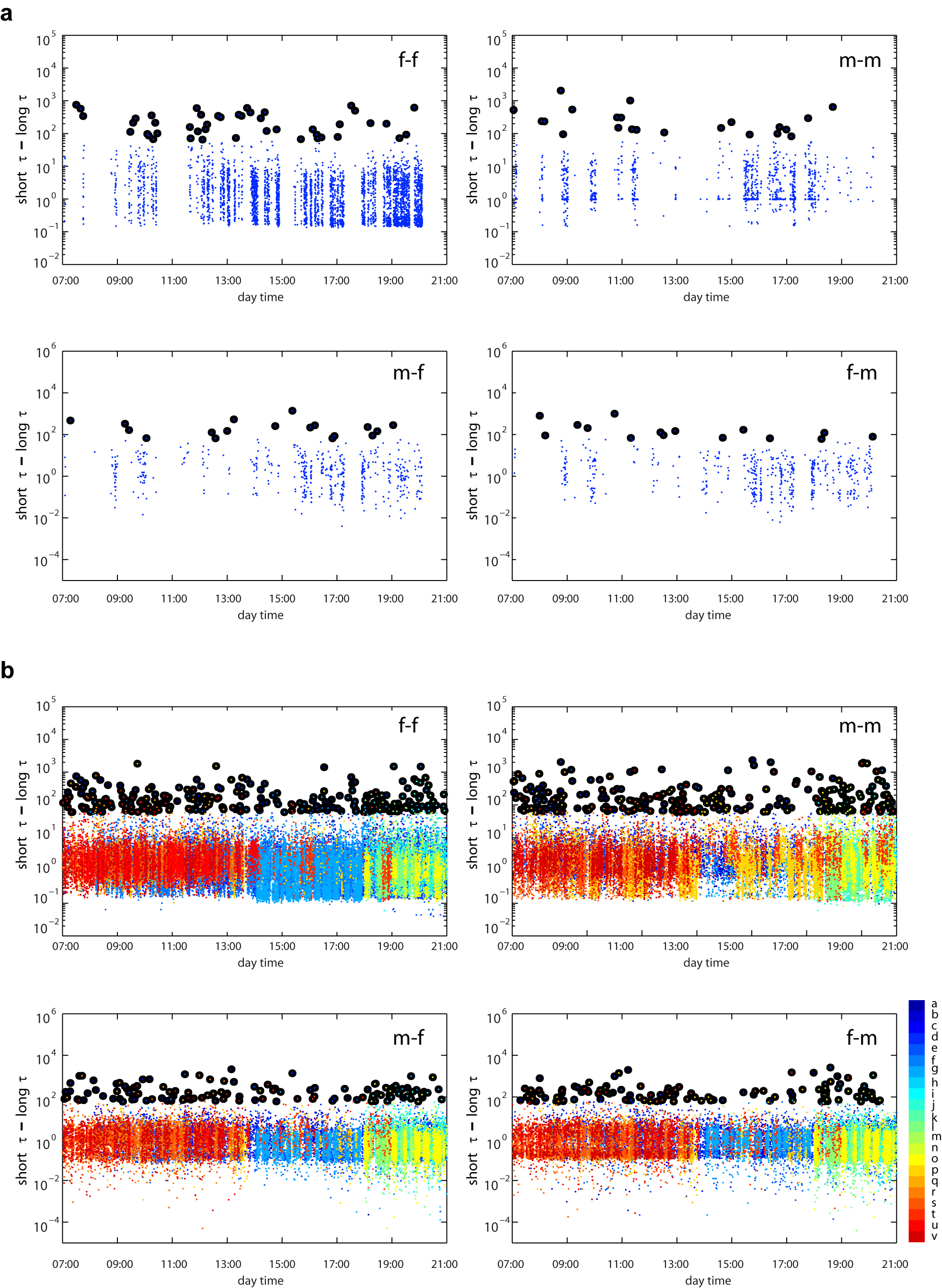


**Supplementary Fig. S5.** Power-law dynamics of calling intervals is not affected by the daily circadian cycle. (**a**) Events of self-consistent (‘f-f’, ‘m-m’) and of reactive (‘m-f’ and ‘f-m’) callings of one zebra finch pair are sorted with respect to their inter-event intervals (*τ*) and plotted against the day time of occurrence. The occurrences of calling events after short and long inter-event intervals (*τ*) distributed equally on the logarithmic scale of inter-event intervals (*τ*) during active periods. (**b**) In all studied pairs of zebra finches, the occurrences of calling events after short *τ* and long *τ* distributed equally on the logarithmic scale of *τ*. Color assignment to 22 pairs of zebra finches (a – v). The events that occurred after long *τ* (> 60 s) are indicated by black circles.

**Supplementary Figure S6**

**
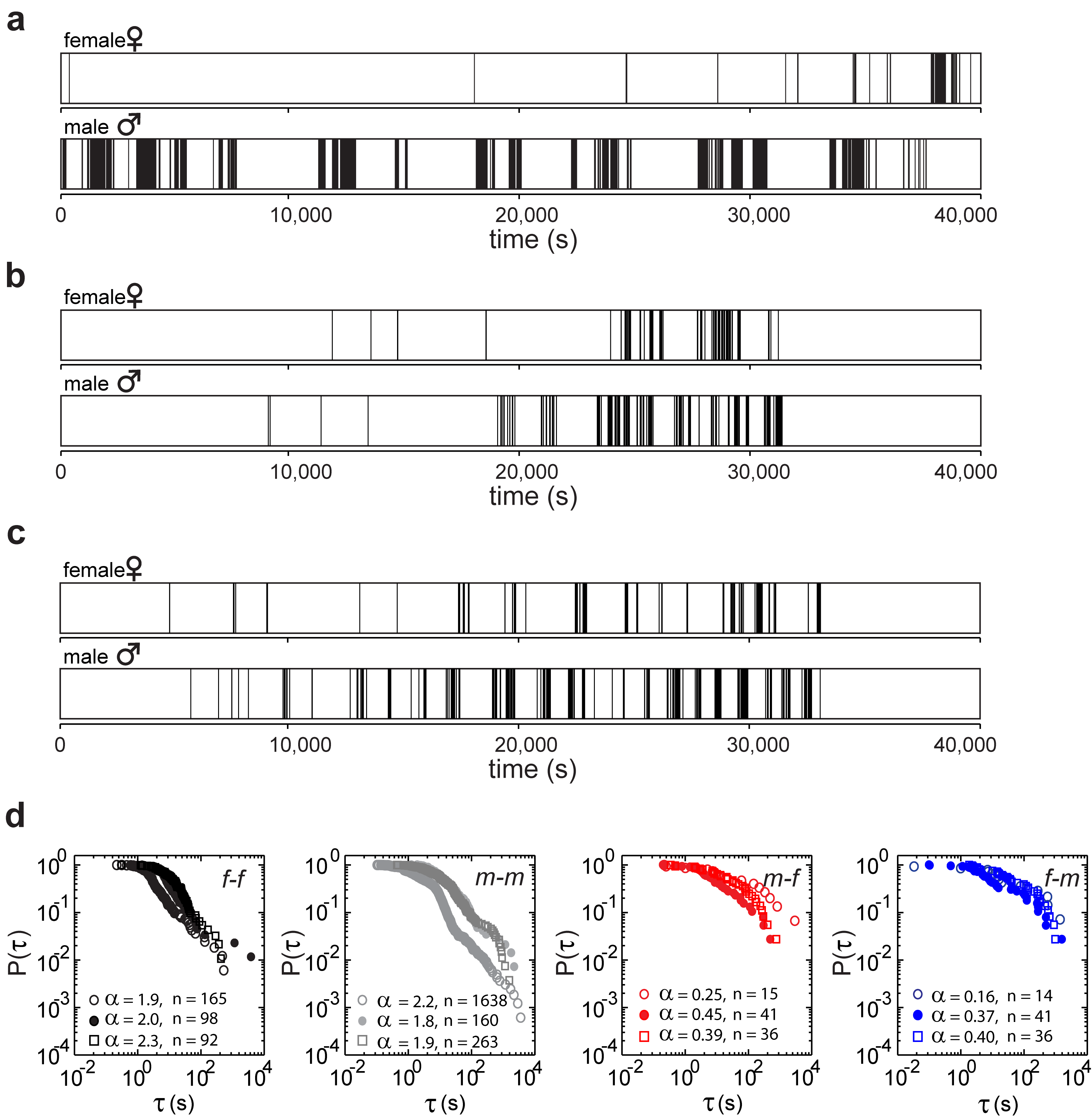
**

**Supplementary Fig. S6. Isolation changes the exponents of “reactive” callings.** (**a - c**): Successions of calling events of three zebra finches pairs after separation of the male and the female of each pair into separated chambers that did not allow visual or vocal interactions with others.. (**d**) Comparision of the empirical cumulative distribution functions (eCDF) and the exponents (α) between τ_f-f_, τ_m-m_, τ_m-f_ and τ_f-m_ of these three zebra finches pairs. Symbol “o”, “•” and “” represent the data of pair a, b and c depicted in (a), (b) and (c), respectively.
